# Tumor-Related Factors Affecting the Success of Interventional Bronchoscopy in Malignant Airway Obstructions

**DOI:** 10.34172/aim.2023.59

**Published:** 2023-07-01

**Authors:** Melahat Uzel Şener, Ayperi Öztürk, Figen Öztürk Ergür, Aydın Yılmaz

**Affiliations:** ^1^Department of Interventional Pulmonology, Ankara Atatürk Chest Diseases and Thoracic Surgery Training and Research Hospital, University of Health Sciences, Ankara, Turkey

**Keywords:** Endobronchial treatment, Malignant, Obstruction, Success, Tumor

## Abstract

**Background:**

It is difficult to select patients who will benefit from endobronchial treatment (ET) in malignant central airway obstruction (MCAO). We aimed to determine the tumor-related factors that affect the success of MCAO treatment.

**Methods:**

ETs for MCAO between March 2019 and June 2021 were analyzed retrospectively. The relationships between the success of the procedure and the percentage of endoluminal obstruction, tumor size, and type of lesion were evaluated.

**Results:**

Totally, 220 ETs were administered to 205 patients. Treatment was significantly more successful for the patients with pure endobronchial lesions than those with mixed lesions. The success rate was significantly lower when the tumor size was greater than 54.5 mm and the degree of endoluminal stenosis exceeded 92%; the area under the curve was 0.734 (0.625–0.842; *P*=0.001) and 0.733 (0.597– 0.870; *P*=0.001), respectively. There was no difference in the procedural success between lung cancer and extrathoracic malignancies and tumor treatment before the procedure.

**Conclusion:**

Mixed lesions, tumor size over 54.5 mm, and a degree of stenosis over 92% are risk factors for unsuccessful endoluminal obstruction procedures. These parameters should be considered when selecting patients for ET interventions.

## Introduction

 Malignant central airway obstruction (MCAO) is airway stenosis that develops in the trachea, main bronchi, intermediate bronchus, and lobar bronchi due to primary or metastatic tumors. Significant dyspnea is observed in patients with stenosis greater than 50% in the lumen.^1,2^

 In treatment of MCAO, there is no standard approach; therefore, differences may exist in endobronchial treatment (ET) depending on the experience of bronchoscopists and the equipment available at centers. In MCAO, interventional bronchoscopy can be applied as a life-saving method in patients with a known diagnosis and/or to improve the quality of life or for both diagnosis and treatment by allowing a biopsy sample to be taken safely. ET may create opportunities for further treatment in patients with MCAO as it may act as a bridge treatment before chemotherapy, radiotherapy, and/or surgery.^3,4^

 Computed tomography (CT) is the first choice for evaluating lesion localization, extraluminal tumor size, and proximity to vascular structures in MCAO and is important regarding patient selection for ET.^5^ The techniques used in ET can be summarized as either hot and cold ablation techniques or mechanical tumor resection (MTR) and dilatation.^6^ Performing the procedure under general anesthesia is safer because it provides airway patency, faster tumor resection, and ease of use of probes.^7^

 Tumor size, the presence of a primary tumor, and dyspnea severity are important when considering ET for patients with MCAO. In our study, we aimed to evaluate the tumor-related factors affecting the success of the procedure in patients who underwent ET due to MCAO. The relationships between the success of the procedure and tumor size, degree of stenosis, radiographic presence of atelectasis before the procedure, and the presence of a primary or metastatic tumor were investigated.

## Materials and Methods

 Our study was planned retrospectively, and approval was obtained from the local ethics committee of the center where the study was conducted. The study data were obtained from the hospital information management system. Patients with MCAO who underwent ET with interventional bronchoscopy between March 2019 and June 2021 were included in the study. Patients with procedures performed due to benign airway stenosis, foreign body aspiration, and post-intubation tracheal stenosis were excluded.

 The patients’ demographic features (i.e., age, gender, and comorbidities), known diagnoses and treatments for malignancy before interventional bronchoscopy, complications related to the procedure, and pathological diagnoses after the procedure were recorded.

###  Interventional Procedures and Definitions

**Figure 1:**
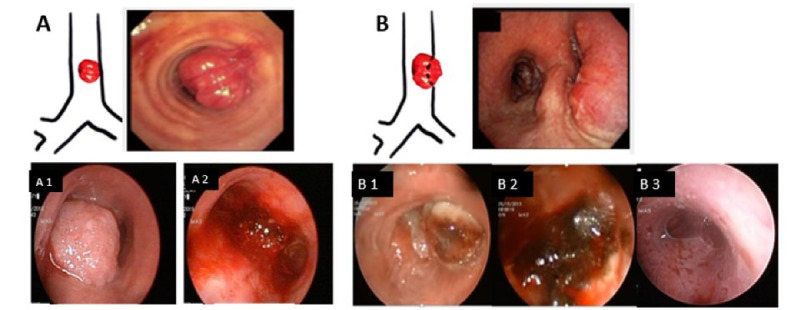
**(A) **Endoluminal lesion, **A1:** Tracheal lesion (before procedure), **A2: **Tracheal lesion (after de-bulking), **(B)** Mixed lesion, **B1: **Right main bronchus obstruction (before procedure), **B2:** Right main bronchus obstruction (after argon plasma coagulation and de-bulking), **B3:** Stent insertion

 All the patients were intubated with a rigid bronchoscope (11 mm diameter, 43 cm length; Efer-Dumon, Efer Endoscopy, Marseille, France) under total intravenous general anesthesia before the procedure was performed. Argon plasma coagulation (APC) (rigid APC probe, 50 cm length, 2.3 mm diameter; ERBE ICC 200/APC 300 electrosurgical unit, ERBE, Germany), MTR, cryoextraction (rigid cryoprobe, 3 mm diameter, 53 cm length; ERBOKRYO CA unit; ERBE, Medizintechnik GmbH, Tübingen, Germany), dilatation, stent placement, and different combinations of these procedures were applied (Figure 1, all parts).

 The anatomical localization of the MCAO and the ratio of the diameter of the intraluminal part of the lesion forming the stenosis to the diameter of the bronchus or trachea in the stenosis localization were recorded as the percentage obstruction. These ratios were divided into 3 groups: less than 50%, 50%–75%, and more than 75%.

 The lesions were categorized as endoluminal lesions and mixed lesions. Lesions with external pressure due to both endoluminal and extraluminal lesions were defined as mixed lesions (Figure 1, A and B). No patients were operated on solely because of extraluminal lesion pressure.

 The long axis of the tumor was recorded as the tumor size via CT before the procedure. Tumor size was divided into 4 groups according to the TNM staging system of the IASLC (T1: ≤ 3 cm, T2: > 3 cm ≤ 5 cm, T3: > 5 cm ≤ 7 cm, T4: > 7 cm).^8^

 The presence of lobar or total atelectasis secondary to MCAO was noted in the chest radiographs taken before the procedure. The patients with atelectasis were controlled via chest X-rays after the procedure.

 The outcomes of the interventions were categorized into 3 groups: successful, partially successful, and unsuccessful. The procedure was classified as successful if the MCAO was almost completely opened, distal airway patency was achieved, intact segment orifices were observed, and atelectasis improved after the procedure. The procedure for the group in which ventilation was provided in the area with atelectasis was recorded as partially successful, while the procedure for the group in which the obstruction could not be opened and atelectasis did not improve was recorded as unsuccessful.

 Cases of mortality during the procedure and in the first 72 hours after the procedure were recorded as procedural mortality.

 The relationships between the procedural success and the tumor size, percentage of obstruction, type of lesion, pre-procedural treatment, presence of atelectasis, and type of malignancy were examined. To assess the probability of a failed intervention, the partially successful and successful groups were combined as a single group and compared with the unsuccessful group. Cut-off values were determined for these two groups for stenosis degree and tumor size parameters.

###  Statistical Analysis

 Statistical analyses were performed using IBM SPSS for Windows version 16.0. Comparisons of the percentages of categorical data were performed using Pearson’s chi-square test and Fisher’s exact test; Fisher’s Exact test was used if the cells with an expected count of less than 5 were greater than 20%. Normality of the continuous data was determined using the Shapiro–Wilk test and Q-Q plot graphs. The Mann–Whitney U test was used for the analysis of the median of the data that was not normally distributed, and the Bonferroni correction was used to analyze the subgroups. An ROC analysis was performed to predict the procedure success in terms of the tumor size and obstruction percentage. A *P* value less than 0.05 indicated statistical significance.

## Results

 In total, 205 patients who underwent ET for MCAO were included in the study. Since the procedure was repeated in 15 patients, 220 interventional procedures were ultimately performed. The mean age of the patients was 62 ± 11 years.

 In terms of the MCAO locations, 20.0% were in the trachea, 3.6% in the main carina, 32.7% in the right main bronchus, 10.5% in the right intermediate bronchus, and 33.2% in the left main bronchus. In 54.1% of the patients (n = 119), the diagnosis was unknown before the procedure, and an interventional procedure was performed for both diagnosis and treatment. In this group, lung cancer was detected in 116 patients (97.5%) and extrathoracic malignancy in 3 patients (2.5%) after the procedure. Of the remaining patients, 38.2% had lung cancer and 7.7% had extrathoracic malignancy. While 140 of the patients (63.6%) had not received any treatment for cancer before the procedure, 80 patients had received chemotherapy, radiotherapy, chemoradiotherapy, surgery, or targeted therapy. The interventional procedures were mostly applied as a combination of hot and mechanical methods (Table 1).

**Table 1:** Main Parameters

		**No. (%)**
Gender	Female	31 (14.1)
	Male	189 (85.9)
Treatment before procedures	None	140 (63.6)
	Chemotherapy	39 (17.7)
	Radiotherapy	4 (1.8)
	Chemoradiotherapy	15 (6.8)
	Surgery	13 (5.9)
	Targeted therapy	4 (1.8)
	Surgery + chemoradiotherapy	5 (2.3)
Pre-procedure atelectasis	None	123 (55.9)
	Yes	97 (44.1)
Post-procedure atelectasis	None	123 (55.9)
	Re-opened	79 (35.9)
	Not-opened	18 (8.2)
Lesion types	Endoluminal	105 (47.7)
	Mixed	115 (52.3)
Procedural success	Successful	158 (71.8)
	Partially successful	45 (20.5)
	unsuccessful	17 (7.7)
Procedures	MTR, APC	145 (65.9)
	Cryoextraction	10 (4.5)
	APC, MTR, cryoextraction	45 (20.5)
	APC, cryoextraction	8 (3.6)
	MTR dilatation	9 (4.1)
	APC, MTR, cryoextraction, stent placement	3 (1.4)
Bleeding	None	163 (74.1)
	Controlled with IV crystalloid fluid	26 (11.8)
	Controlled with APC	30 (13.6)
	Uncontrolled	1 (0.5)
Complications (other than bleeding)	Respiratory failure	1 (0.5)
	Arrhythmia	2 (0.9)
Pathological diagnosis	Squamous	118 (53.6)
	Adeno	19 (8.6)
	Small	16 (7.3)
	Carcinoid	14 (6.4)
	Adenoid cystic carcinoma	7 (3.2)
	Malignant epithelial tumor	3 (1.4)
	Extrathoracic malignancy	13 (5.9)
	NOS	24 (10.9)
	Others	6 (2.7)

MTR, Mechanical tumor resection; APC, Argon plasma coagulation; IV, Intravenous; NOS, not otherwise specified.

 The pathology results after the procedure were 53.6% squamous cell carcinoma, 10.9% not otherwise specified, 8.6% adenocarcinoma, 7.3% small cell carcinoma, and 5.9% extrathoracic malignancy. Of the 220 procedures, 158 (71.8%) were successful, 45 (20.5%) were partially successful, and 17 (7.7%) were unsuccessful (Table 1).

 Based on the chest X-ray results before the procedure, 97 patients (44.1%) had atelectasis. In 79 of these patients (81.4%), it was observed that the atelectasis regressed after the procedure and ventilation was achieved in the lungs (Table 1). The success of the procedure was significantly higher in the patients without atelectasis before the procedure (*P* < 0.001) (Table 2). When the lesion localizations and procedure success were evaluated, the percentages of successful procedures in the trachea, right main bronchus, right intermediate bronchus and left main bronchus were 100%, 95.8%, 91.3%, 84.9%, respectively (*P* = 0.015). It was found that the groups that differed were “Trachea” and “Left main bronchus” (Bonferroni correction).

**Table 2:** Treatment Modalities, Diagnosis, Lesion Types

		**Procedural Success**			
		**Successful No. (%)**	**Partially Successful No. (%)**	**Unsuccessful No. (%)**	***P *** **Value**^a^
Total		158 (71.8)	45 (20.5)	17 (7.7)	‒
Chemotherapy		36 (61)	16 (27.1)	7 (11.9)	0.089
Radiotherapy		16 (66.7)	5 (20.8)	3 (12.5)	0.577*
Surgery		15 (83.3)	3 (16.7)	0 (0)	0.541*
Pre-procedure atelectasis		65 (67)	16 (16.5)	16 (16.5)	< 0.001
Lesion types	Endoluminal	105 (100)	0 (0)	0 (0)	< 0.001
	Mixed	53 (46.1)	45 (39.1)	17 (14.8)	
Pathological diagnosis	Lung Cancer	149 (72)	43 (20.8)	15 (7.2)	0.425*
	Extrathoracic malignancies	9 (69.2)	2 (15.4)	2 (15.4)	
Pathological diagnosis	Squamous	75 (63.6)	32 (27.1)	11 (9.3)	‒
	Adeno	14 (73.7)	3 (15.8)	2 (10.5)	
	Small	10 (62.5)	5 (31.3)	1 (6.3)	
	Carcinoid	14 (100)	0 (0)	0 (0)	
	Adenoid cystic carcinoma	7 (100)	0 (0)	0 (0)	
	Malignant epithelial tumor	3 (100)	0 (0)	0 (0)	
	Extrathoracic malignancy	9 (69.2)	2 (15.4)	2 (15.4)	
	NOS	20 (83.3)	3 (12.5)	1 (4.2)	
	Others	6 (100)	0 (0)	0 (0)	

^a^ Pearson chi-square test; *Fisher’s exact test.

 In terms of lesions, 105 (47.7%) were pure endoluminal lesions and 115 (52.3%) were mixed lesions. No patient was treated because of external tissue pressure alone. The procedure was successful in three patients with stent implantation due to mixed lesion. The procedures were significantly more successful in the patients with endoluminal lesions (*P* < 0.001) (Table 2).

 All the patients were treated for MCAO, and three of them had hemoptysis before the procedure. No complications occurred in 160 patients during the procedure, bleeding developed in 57 patients, and hemorrhage was controlled with saline in 26 patients and APC in 30 patients. Bleeding control could not be achieved in one patient, and the patient died intraoperatively. Respiratory failure developed in one patient, and arrhythmia developed in two patients. With the exception of the patient who died during the procedure, no deaths were observed in the first 72 hours following the procedure.

 When the relationships between the treatments received before the procedure and the success of the procedure were examined, no differences were apparent in terms of success in the patients who underwent chemotherapy, radiotherapy, or surgical treatment. Although the highest rate of unsuccessful procedures was observed in those with extrathoracic malignancies (15.4%), the difference was not statistically significant (Table 2).

 The success rates by cancer subtype are shown in Table 2. The success rates were similar in the patients with squamous and small cell carcinoma but higher in those with adenocarcinoma. All the procedures were successful in the patients with carcinoid tumor, adenoid cystic carcinoma, and malignant epithelial tumor.

 When the interventions were divided into groups, the mean obstruction percentages in the successful, partially successful, and unsuccessful groups were 79% ± 24%, 70% ± 25%, and 90% ± 21%, respectively (*P* < 0.001), and the mean tumor sizes were 54 ± 28 mm, 68 ± 27 mm, 82 ± 30 mm, respectively (*P* < 0.001).

 The endoluminal obstruction percentage was divided into 3 groups (i.e., < 50%, 50%–75%, ≥ 75%). A statistical difference was found across these groups in terms of procedural success. In the group with 50%–75% obstruction, 44.1% of the procedures were partially successful. The rate of treatment success was significantly reduced in the patients with tumors larger than 30 mm. The success rates of the procedures were found to be similar in the patients with tumors of 50–70 mm and ≥ 70 mm in size (Table 3).

**Table 3:** Obstruction Grades, Tumor Size

		**Procedural Success **			
		**Successful No. (%)**	**Partially Successful No. (%)**	**Unsuccessful No. (%)**	***P *** **Value**^a^
Grade (obstruction)	< 50	25 (73.5)	8 (23.5)	1 (2.9)	0.004
	≥ 50 and < 75	17 (50)	15 (44.1)	2 (5.9)	
	≥ 75	116 (76.3)	22 (14.5)	14 (9.2)	
Tumor size	< 30	34 (97.1)	1 (2.9)	0 (0)	0.001
	≥ 30 and < 50	38 (77.6)	10 (20.4)	1 (2)	
	≥ 50 and < 70	42 (65.6)	14 (21.9)	8 (12.5)	
	≥ 70	44 (61.1)	20 (27.8)	8 (11.1)	

^a^Fisher’s exact test. The difference according to the percentage of stenosis is due to the Grade-2 ( ≥ 50 and < 75) group. The difference according to tumor size is due to the 1st stage ( < 30) group.

**Figure 2:**
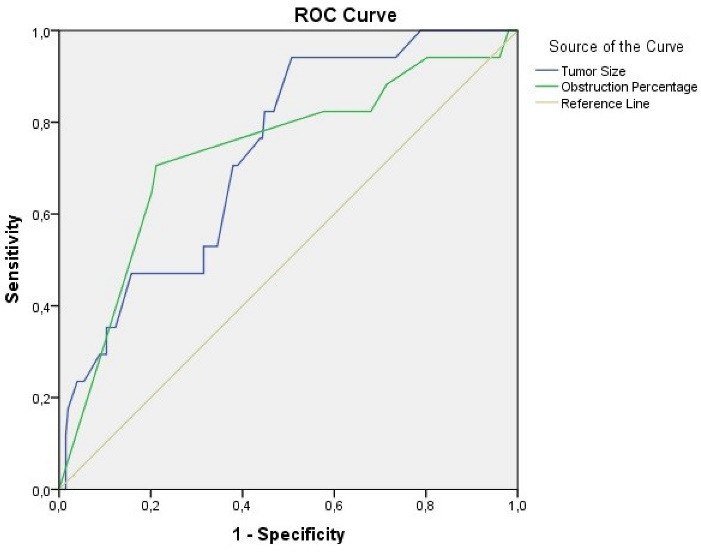
ROC Curve for Tumor Size and Obstruction Percentage According to Procedure Success

 Successful and partially successful groups were combined (Table 4) and ROC analysis was performed for the final two groups (successful and unsuccessful). In this analysis to evaluate the effects of tumor size and obstruction percentage on the success of the procedure, the area under the curve was found to be 0.734 (0.625–0.842) and 0.733 (0.597–0.870), respectively (Figure 2). The sensitivity and specificity of these two parameters for predicting failed procedures are presented in Table 5. The highest Youden index values were found to be 54.5 mm for tumor size and 92.5% for obstruction percentage.

**Table 4:** Obstruction Grades and Tumor Size in Two Groups

	**Successful or Partially Successful (n=203)**		**Unsuccessful (n=17)**		***P *** **Value**^a^
	**Mean±SD**	**Median (IQR)**	**Mean±SD**	**Median (IQR)**	
Tumor size	57 ± 28	55 (35‒76)	82 ± 30	69 (60‒100)	0.001
Obstruction percentage	77 ± 24	90 (60‒90)	90 ± 21	100 (90‒100)	0.001

^a^ Mann-Whitney U test.

**Table 5:** Coordinates of the Curve

**Test Result Variable(s)**	**Positive if≥**	**Sensitivity (%)**	**Specificity (%)**	**Youden Index**
Tumor size (mm)	54.5	94.1 (71.3‒99.9)	49.3 (42.2‒56.4)	0.434
	57.5	82.4 (56.6‒96.2)	55.2 (48.1‒62.1)	0.376
	63.5	70.6 (44.0‒89.7)	62.1 (55.0‒68.8)	0.327
	68.5	52.9 (27.8‒77.0)	68.5 (61.6‒74.8)	0.214
	87.5	47.1 (23.0‒72.2)	84.2 (78.5‒89.0)	0.313
Obstruction percentage (%)	75.0	82.4 (56.6‒96.2)	32.0 (25.7‒38.9)	0.144
	87.5	82.4 (56.6‒96.2)	42.4 (35.5‒49.5)	0.248
	92.5	75.0 (50.9‒91.3)	78.8 (72.6‒84.2)	0.494
	97.5	64.7 (38.3‒85.8)	79.8 (73.6‒85.1)	0.445

The test result variable(s): Tumor size, obstruction percentage has at least one tie between the positive actual state group and the negative actual state group. a. The smallest cutoff value is the minimum observed test value minus 1, and the largest cutoff value is the maximum observed test value plus 1. All the other cutoff values are the averages of two consecutive ordered observed test values.

## Discussion

 No clear algorithm exists to determine success in ET for MCAO, and it is difficult to select patients who will benefit from the procedure. In our study, we investigated the effect of tumor-related factors on the success of ET. The success rate for mixed lesions was significantly lower in lesions with an endoluminal obstruction grade above 92% and/or a tumor size over 54.5 mm. However, there was no difference in procedural success between the treatments applied before the procedure and lung cancer and extrathoracic malignancies.

 Interventional bronchoscopic techniques used in the treatment of airway obstruction include MTR, debridement, stenting, dilatation, and disobliteration of stenotic material with laser, argon plasma, cautery, or cryotherapy.^6,9^ Despite the potential benefits of palliation of symptomatic airway obstruction, there is still a large gap in the management of patients with malignant obstruction as these patients are perceived to have particularly poor outcomes.^9^

 Lung cancer may be complicated by 20%–30% proximal airway obstruction, which is usually seen in advanced disease.^10^ Endobronchial metastases of other solid tumors are rarer and usually occur late after diagnosis.^11^ There is no technical difference in the approach of ET for lung cancer and extrathoracic malignancy. In our study, no statistically significant difference was evident between lung cancer and extrathoracic malignancies in terms of procedural success. Similar to our study, Giovacchini et al found that primary cancer type or lung cancer cell subtype had no effect on the success of the procedure.^12^

 Bronchoscopy and CT of the thorax are recommended for patient selection before the procedure to evaluate the endoluminal extension of the lesion, the presence and compression of the extraluminal tumor, and the continuation of the airway patency distal to the lesion.^13^ In our study, tumor size was measured using thorax CT before the procedure. We observed that the success rate of the procedure decreased as the tumor size increased, and we found that 54.5 mm was an important threshold value in this respect. To the best of our knowledge, this is the first study in the literature to examine the relationship between tumor size and the success of an interventional procedure.

 Giovacchini et al evaluated the factors affecting the technical success of therapeutic bronchoscopy and found that pre-procedural patent distal airway monitoring with CT and flexible bronchoscopy were independent factors influencing the success of the procedure.^12^ In a multicenter study by Ost et al, lung cancer and left system involvement were found to be risk factors for unsuccessful procedures. In addition, pure endobronchial obstruction was found to be associated with technical success.^14^ Hespanhol et al created a success prediction model and found that tracheal location, pure endobronchial disease, and external compression predicted positive procedural results, while left main root obstruction and mucosal infiltration of the tumor were associated with reduced probability of success.^15^ In our study, a significant difference was found between endoluminal lesions and mixed lesions in terms of success, with a 100% success rate for endobronchial lesions. The success rate (84%) was found to be the lowest for left main bronchus involvement. All the procedures were successful for tracheal involvement.

 In studies based on endobronchial tumor size in the literature, the most suitable patients for curative treatment were those with an endoluminal part less than 10 mm and without an extra cartilage component.^16^ In the study by Yilmaz et al, the degree of endoluminal stenosis did not have a significant effect on the success of the procedure, but the success rate was significantly lower in the patients with distal bronchial involvement.^17^ It has been stated that interventional bronchoscopy is not an appropriate procedure in the presence of distal bronchial tree lesions or airless parenchyma, and other treatment modalities (such as chemotherapy) should be applied first in these patients.^18^ In the case of obstruction at a lobar level, bronchoscopic approaches are indicated only to control hemoptysis or post-obstructive pneumonia drainage as ventilation is usually not significantly improved in these patients.^19^ In our clinical experience, drainage of post-obstructive pneumonia is very important for the continuation of other treatments for malignancies. After successfully controlling the infection, it may be safe to switch to systemic therapies. Accordingly, the patients in our study in whom distal segment continuity was not observed during the procedure but whose lung tissue was re-aerated were considered to have had partially successful procedures because of the chance of bridge treatment. Since the procedural risk is high for this patient group, the benefit–harm relationship should be considered during decision-making.

 There are few studies in the literature to have addressed the tumor-related factors affecting the success of interventional procedures, but they investigated the factors affecting post-procedural prognoses in MCAO. In a retrospective cohort, receiving post-procedural chemotherapy was found to be an independent prognostic factor for survival.^9^ In another study, post-interventional chemotherapy or radiotherapy was found to be a good prognostic factor.^4^ In our study, neither chemotherapy, radiotherapy, nor surgery before the procedure had any significant effects on the success of the procedure. This should be taken into account in decision-making regarding interventions.

 In our study, procedure-related mortality was seen in a single patient. In our opinion, this rate (0.5%) is low even though the majority of the patients were severely symptomatic and debilitated. In the study by Guibert et al, mortality rate due to the procedure reached 1.9% only in those with metastatic disease whose general conditions were extremely changed; however, survival in the previously treated patients was significantly better than that of the untreated patient group.^20^

 The main limitation of our study was its retrospective design. CT was examined in all patients before the procedure, but since the data were obtained from the hospital database, we were unable to examine the initial diagnostic radiological images at the time of diagnosis of some patients who were referred to our hospital for interventional procedure from other centers. Therefore, there was lack of information about the airway status at the time of diagnosis in these patients. In prospective studies, symptom evaluations of patients before and after the procedure, the treatments received after the procedure, and other indicators of procedural success in terms of survival can be analyzed. The fact that there were 17 cases in the unsuccessful group also reduces the reliability of the statistical analyses between the two groups.

## Conclusion

 In conclusion,tumor-related factors should be evaluated during patient selection for interventional bronchoscopic treatment in patients with MOAC. In our study, diagnoses of lung cancer and extrathoracic malignancy and treatments for existing cancers did not have any effect on the success of the procedure. In patients with mixed lesions, the rate of unsuccessful procedures increased in the presence of atelectasis before the procedure. It is understood that the percentage of endoluminal obstruction and tumor size measured on CT can affect success. The success rate in our study was severely reduced in the patients with lesions with an obstruction percentage greater than 92% and/or a tumor size of more than 54.5 mm. It is necessary to pay particular attention to these parameters when selecting the appropriate patients for interventional bronchoscopic treatment.
